# Response Shift After Cognitive Behavioral Therapy Targeting Severe Fatigue: Explorative Analysis of Three Randomized Controlled Trials

**DOI:** 10.1007/s12529-022-10111-8

**Published:** 2022-07-22

**Authors:** Fabiola Müller, Mathilde G. E. Verdam, Frans J. Oort, Heleen Riper, Annemieke van Straten, Irma M. Verdonck-de Leeuw, Mirjam A. G. Sprangers, Hans Knoop

**Affiliations:** 1 Medical Psychology, Amsterdam UMC Location University of Amsterdam, Meibergdreef 9, Amsterdam, The Netherlands; 2 Amsterdam Public Health, Global Health, Amsterdam, The Netherlands; 3 Amsterdam Public Health, Mental Health, Amsterdam, The Netherlands; 4 Cancer Center Amsterdam, Cancer Treatment and Quality of Life, Amsterdam, The Netherlands; 5 Faculty of Science, School of Psychology, Quality of Life Office, The University of Sydney, Chris O’Brien Lifehouse (C39Z), Sydney, NSW 2006 Australia; 6 Amsterdam Public Health, Personalized Medicine, Amsterdam, The Netherlands; 7 Institute of Psychology, Department of Methodology and Statistics, Leiden University, Pieter de la Court Building, Wassenaarseweg 52, 2333 AK Leiden, The Netherlands; 8 Research Institute of Child Development and Education, University of Amsterdam, Nieuwe Achtergracht 127, 1018 WS Amsterdam, The Netherlands; 9 Department of Clinical, Neuro-, & Developmental Psychology, Faculty of Behavioural and Movement Sciences, Vrije Universiteit Amsterdam, Van der Boechorststraat 7, 1081 BT Amsterdam, The Netherlands; 10 Department of Psychiatry, Amsterdam UMC Location Vrije Universiteit Amsterdam, De Boelelaan 1117, Amsterdam, The Netherlands; 11 Department of Otolaryngology-Head and Neck Surgery, Amsterdam UMC Location Vrije Universiteit Amsterdam, De Boelelaan 1117, Amsterdam, The Netherlands; 12 Aging & Later Life, Amsterdam Public Health, Amsterdam, The Netherlands; 13 Expert Center for Chronic Fatigue, Amsterdam UMC Location Vrije Universiteit Amsterdam, De Boelelaan 1117, Amsterdam, The Netherlands

**Keywords:** Cognitive behavioral therapy, Fatigue, Response shift, Checklist Individual Strength, Structural equation modelling

## Abstract

**Background:**

Cognitive behavioral therapy (CBT) is an evidence-based intervention for severe fatigue. Changes in patients’ fatigue scores following CBT might reflect not only the intended relief in fatigue but also response shift, a change in the meaning of patients’ self-evaluation. Objectives were to (1) identify the occurrence of response shift in patients undergoing CBT, (2) determine the impact of response shift on the intervention effect, and (3) investigate whether changes in fatigue-related cognitions and perceptions, targeted during CBT, are associated with response shift.

**Methods:**

Data of three randomized controlled trials testing the efficacy of CBT in individuals with chronic fatigue syndrome (CFS, *n* = 222), cancer (*n* = 123), and diabetes (*n* = 107) were re-analyzed. Fatigue severity was measured with 8 items from the Checklist Individual Strength, a valid and widely used self-report questionnaire. Structural equation modelling was applied to assess lack of longitudinal measurement invariance, as indication of response shift.

**Results:**

As expected, in all three trials, response shift was indicated in the CBT groups, not the control groups. Response shift through reprioritization was indicated for the items “Physically, I feel exhausted” (CFS) and “I tire easily” (cancer, diabetes), which became *less* vs. *more* important to the measurement of fatigue, respectively. However, this did not affect the intervention effects. Some changes in cognitions and perceptions were associated with the response shifts.

**Conclusions:**

CBT seems to induce response shift through reprioritization across patient groups, but its occurrence does not affect the intervention effect. Future research should corroborate these findings and investigate whether patients indeed change their understanding of fatigue.

**Supplementary Information:**

The online version contains supplementary material available at 10.1007/s12529-022-10111-8.

## Introduction

Fatigue is a highly distressing and interfering symptom that is common among individuals with chronic conditions. Severe fatigue is the principal symptom in individuals with chronic fatigue syndrome (CFS) [1, 2] and is experienced by approximately 30% of individuals treated for cancer [3, 4] and around 40% of individuals with type 1 diabetes [5, 6]. Fatigue experienced by these patients is substantially different from everyday fatigue, as the former is an unpleasant physical and mental sensation that persists for months and substantially interferes with patients’ functioning [7].

Cognitive behavioral therapy (CBT) is an evidence-based intervention aimed at reducing fatigue severity among patients with chronic conditions. CBT is based on the cognitive-behavioral model of fatigue, which states that disease and its treatment initially precipitate fatigue, while cognitive and/or behavioral variables perpetuate fatigue in the long-term [6, 8, 9]. The fatigue-perpetuating variables are largely transdiagnostic, that is, explain fatigue across various chronic conditions [10]. Accordingly, CBT intervenes upon these transdiagnostic perpetuating cognitive-behavioral variables in addition to disease-specific perpetuating variables, such as fear of cancer recurrence among cancer survivors. CBT has been found to reduce fatigue in a range of chronic conditions, including CFS [11, 12] and cancer [13, 14]. Treatment effectiveness is commonly evaluated through assessing changes in patient’s self-reported fatigue. A reduction in self-reported fatigue from pre- to post-treatment is taken to indicate a positive intervention effect: a reduction in fatigue severity. However, as CBT also directly targets patients’ cognitions about fatigue, it may also induce a change in the meaning that patients attach to their fatigue evaluation, which is also known as response shift.

**Fig. 1:**
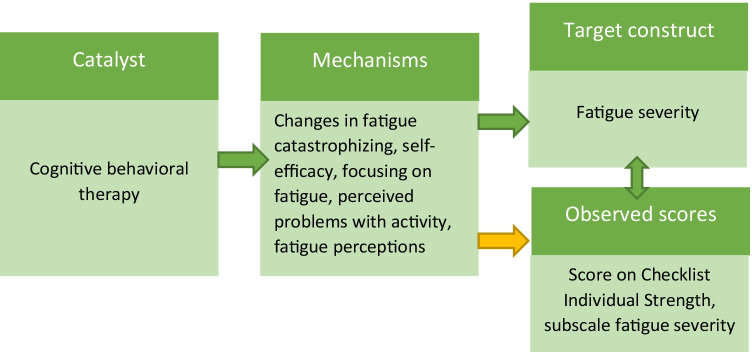
Theoretical model of response shift as applied to the current study. Note: The arrow from 'Mechanisms' to 'Observed scores' (in yellow) signifies response shift

Response shift relies on the distinction between the *observed scores* (e.g., scores on a fatigue questionnaire) and the *target construct* (e.g., fatigue itself) and a possible change in their association over time [15]. Accordingly, response shift is defined as a discrepancy between measured change (e.g., a decreased score on a fatigue questionnaire) and change in the target construct (e.g., a decrease in fatigue severity) that is due to a change in the meaning of one’s self-evaluation. For example, a decrease in the score on a fatigue item from “6” to “4” may occur because the meaning of the score “6” is no longer the same after treatment as it was before treatment, and not because there is a decrease in fatigue severity. Following the theoretical models of response shift [15, 16], a catalyst is assumed to initiate this process: the catalyst (e.g., CBT targeting fatigue) induces change in an individual’s health that may trigger psychological mechanisms (e.g., cognitive adaptations) to accommodate health change, which may not only affect target change (e.g., change in fatigue severity) but may also induce response shift (see Fig. 1). Response shift can occur, for example, through a change in (1) patients’ internal standards with which they assess their fatigue (i.e., “recalibration”), (2) the relative importance patients assign to different aspects of fatigue (i.e., “reprioritization”), or (3) the meaning of fatigue itself (i.e., “reconceptualization”) [16]. Importantly, when response shift occurs, this may render the comparison of self-evaluations over time incompatible.

Response shift has been studied in patients who face a catalyst that triggers either a (temporal) deterioration in patients’ health status (e.g., toxic cancer treatment or disease progression) or improvement in their health (e.g., intervention aimed at symptom relief). These studies provided evidence that response shift in the measurement of self-reported health outcomes, including fatigue, can occur [17–21] and that it may be triggered by an intervention such as CBT [22]. These studies also indicate that the occurrence of response shift, if not taken into account, can lead to biased conclusions — either over- or underestimation — about the health impact of the catalyst [19, 20, 23, 24]. While acknowledging that eliciting a re-evaluation of fatigue might also be a clinically meaningful intervention effect [24], response shift that is not accounted for might result in false conclusions about the efficacy of the intervention and, hence, suboptimal patient care [25]. Consequently, gaining insight into the occurrence and impact of response shift is of both clinical and methodological relevance: First, it helps to understand how CBT reduces fatigue (i.e., by decreasing the actual level of fatigue severity and/or by helping patients to re-evaluate their fatigue level). Second, it can indicate whether the possible occurrence of response shift might change our interpretation of the CBT-effect.

In line with the cognitive-behavioral model of fatigue, CBT aims to change patients’ dysfunctional cognitions assumed to perpetuate fatigue, including fatigue catastrophizing, negative expectations and feelings of helplessness regarding fatigue, low self-efficacy, the extent to which patients think they are able to influence fatigue, and an extensive focus on fatigue. Also, patients’ activity-related cognitions are addressed as patients are challenged to gradually increase their daily activities. Improvements in these fatigue- and activity-related cognitions appear to play a mediating role in the reduction in fatigue following CBT [10, 26, 27]. Additionally, changes in patients’ perceptions of fatigue, such as it being exhausting or pleasant, appear to accompany the decrease in fatigue severity following CBT [28, 29]. Improvements in these cognitions and perceptions may not only explain the reduction in patients’ fatigue severity but may also explain patients’ new evaluation of their fatigue.

In the current paper, data of three randomized controlled trials (RCTs) were re-analyzed, in which CBT was found to be effective in relieving severe fatigue, as compared to a control group [30–32]. The first objective was to identify the occurrence of response shift in patients undergoing CBT for severe fatigue. Response shift was expected to occur in the CBT group only, as theory and evidence suggest that a catalyst, such as an effective intervention, is required to induce response shift [15, 16, 33]. CBT directly targets cognitions regarding fatigue. It was therefore assumed to be likely that CBT can affect the meaning of patients’ subjective evaluation of fatigue. There were no expectations regarding how response shift may occur (i.e., through recalibration, reprioritization, or reconceptualization). If response shift occurred, the second objective was to determine its impact on the intervention effect of CBT. As response shift caused by CBT would impact how participants interpret and respond to the fatigue questionnaire at the post-intervention assessment, it was expected that taking its occurrence into account would alter the estimated intervention effect of CBT. As there were no expectations regarding how response shift might occur, there were also no expectations regarding the direction of the effect (i.e., over- or underestimation). The third objective was to investigate whether changes in fatigue-related cognitions and perceptions, targeted during CBT, are associated with the occurrence of response shift. Investigating these associations is hoped to inform whether changes in these cognitions and perceptions are possible mechanisms of response shift. It was expected that a reduction in catastrophizing, focusing on fatigue, problems with activity and negative perceptions of fatigue and an increase in self-efficacy and positive perceptions of fatigue would be associated with detected response shift.

To address the three objectives, Oort’s widely used structural equation modelling (SEM) approach [34, 35] was applied. SEM allows for modelling the relations between the observed scores on the fatigue questionnaire and the underlying target construct (i.e., fatigue severity). Response shift effects are operationalized using the concept of measurement (non)invariance [36]. A lack of measurement invariance over time indicates that observed change on the fatigue questionnaire is not only due to change in fatigue severity but also due to change in the relationship between the observed scores and fatigue severity. The SEM framework for response shift detection is innovative as it formulates a direct link between these statistical operationalizations of measurement invariance and the conceptualizations of change due to response shift effects. The three types of response shift as defined by Sprangers and Schwartz [16], i.e., recalibration, reprioritization, and reconceptualization, are operationalized through change in specific model parameters. In the recently proposed revised response shift model by Vanier and colleagues [15], the three types are not included in the definition but are acknowledged as possible pathways to response shift. Therefore, rather than referring to the three types of response shift [16], recalibration, reprioritization, and reconceptualization are described as possible pathways through which response shift can occur [15]. Moreover, by including hypothesized psychological mechanisms for response shift into the model, the SEM approach can serve as a useful tool to investigate possible explanations for response shift.

## Method

Individuals with CFS (CFS-trial, [31]), with breast cancer (Cancer-trial, [30]), and with type 1 diabetes (Diabetes-trial, [32]) were randomized to CBT or a control group (waitlist). Patients were eligible if they were severely fatigued as indicated by a score of ≥ 35 on the fatigue severity subscale of the Checklist Individual Strength (CIS-fatigue), were aged ≥ 18 years, and able to speak, read, and write Dutch. Assessments were performed before randomization (pre-assessment) and after the intervention or waiting period (post-assessment), that is, 5 months (Diabetes-trial) or 6 months (CFS- and Cancer-trials) later. All trials were approved by the relevant medical ethical committee and patients provided written informed consent prior to their participation. CBT was conducted according to the cognitive-behavioral model of fatigue and delivered by trained cognitive-behavioral therapists. Patients were offered modules which target perpetuating factors of fatigue. Details about the RCTs are reported in the Electronic Supplementary Material 1.

As the aim is to investigate response shift induced by CBT, patients randomized to the CBT group but who did not start or complete the intervention (*n* = 10 (6%), *n* = 5 (8%), and *n* = 11 (18%), for the CFS-trial, Cancer-trial, and Diabetes-trial, respectively) and patients randomized to the control group but who underwent another evidence-based fatigue intervention (as enquired at the post-assessment; *n* = 0 (0%), *n* = 1 (2%), *n* = 2 (3%), for the CFS-trial, Cancer-trial, and Diabetes-trial, respectively) were excluded from the current analyses. As the chosen analytic technique can only handle complete data, data from eight patients who did not complete the post-assessment were also excluded (*n* = 1 (CBT group), *n* = 4 (control group); *n* = 1 (CBT group), *n* = 2 (control group) from the CFS-trial and Cancer-trial, respectively). See Table 1 for patient characteristics.

**Table 1:** Sample descriptives of three randomized controlled trials testing the efficacy of cognitive behavioral therapy for severe fatigue

	**CFS-trial** Janse et al. [31]		**Cancer-trial** Abrahams et al. [30]		**Diabetes-trial** Menting et al. [32]	
**Patient group**	Chronic fatigue syndrome		Breast cancer		Type 1 diabetes	
**Intervention**	Internet-based CBT		Internet-based CBT		Blended CBT	
**Study design**	3-arm RCT* Pre- and 6 months post-assessment		2-arm RCT Pre- and 6 months post-assessment		2-arm RCT Pre- and 5 months post-assessment	
**Recruitment period**	April 2013 to June 2015		January 2014 to March 2016		February 2014 to March 2016	
**Recruitment strategy**	Via Expert Center for Chronic Fatigue, the Netherlands		Via eight hospitals and self-referral, the Netherlands		Via five hospitals and self-referral, the Netherlands	

	**CBT group** *n* = 149	**Control group** *n* = 73	**CBT group** *n* = 60	**Control group** *n* = 63	**CBT group** *n* = 49	**Control group** *n* = 58
**Age**	37 (13) (range 18–64)	41 (13) (range 19–67)	52 (8) (range 32–72)	50 (8) (range 31–68)	44 (13) (range 20–71)	42 (13) (range 20–70)
**Gender, female, *n* (%)**	91 (61.1%)	41 (56.2%)	60 (100%)	63 (100%)	31 (63.3%)	35 (60.3%)
**Fatigue, pre** (Cronbach’s alpha)	50.29 (5.23) (0.69)	49.66 (5.24) (0.69)	45.22 (7.00) (0.79)	44.71 (7.41) (0.74)	46.14 (6.18) (0.77)	45.97 (5.92) (0.71)
**Fatigue, post** (Cronbach’s alpha)	36.90 (13.82) (0.95)	44.62 (9.88) (0.90)	26.43 (11.06) (0.93)	38.92 (11.12) (0.90)	24.59 (10.47) (0.93)	39.98 (10.39) (0.90)
**FCS, pre**	23.34 (6.91)	23.60 (7.32)	21.35 (5.92)	21.44 (5.94)	21.00 (6.00)	20.83 (6.31)
**FCS, post**	18.04 (6.60)	20.96 (7.36)	15.21 (5.00)	19.72 (5.72)	14.49 (4.25)	18.78 (5.24)
**SES, pre**	16.86 (2.96)	17.14 (2.75)	18.85 (2.81)	18.19 (3.30)	18.43 (2.25)	17.95 (2.58)
**SES, post**	20.30 (4.16)	18.50 (3.00)	22.71 (3.47)	19.07 (3.84)	23.04 (3.71)	19.11 (2.95)
**IMQ, pre**	32.22 (9.66)	32.60 (9.53)	31.87 (8.33)	33.00 (7.91)	26.67 (7.43)	27.12 (8.51)
**IMQ, post**	22.73 (10.03)	27.57 (9.79)	18.26 (8.61)	27.05 (8.50)	18.89 (6.41)	25.29 (8.92)
**ACT, pre**	16.54 (4.18)	16.62 (4.26)	14.62 (4.57)	13.57 (4.98)	13.39 (4.85)	13.38 (5.43)
**ACT, post**	11.78 (5.54)	14.53 (5.03)	9.33 (4.36)	11.98 (4.98)	8.18 (4.51)	12.24 (5.32)
**FQL frustrating, pre**	74.63 (26.98)	79.17 (22.12)	61.67 (24.78)	70.65 (28.22)	60.82 (27.37)	58.97 (28.64)
**FQL frustrating, post**	46.33 (35.22)	73.06 (28.71)	21.03 (29.84)	55.44 (34.23)	14.89 (22.64)	43.64 (32.68)
**FQL exhausting, pre**	55.54 (30.60)	52.08 (30.76)	37.50 (34.29)	39.11 (30.58)	30.10 (31.45)	30.17 (28.00)
**FQL exhausting, post**	24.82 (29.41)	45.49 (35.93)	6.47 (20.17)	26.75 (33.02)	5.85 (14.94)	27.27 (31.28)
**FQL frightening, pre**	36.07 (26.54)	35.76 (21.32)	17.92 (20.63)	20.97 (19.31)	25.00 (21.65)	17.67 (19.31)
**FQL frightening, post**	16.55 (21.82)	25.69 (27.51)	3.02 (9.46)	9.21 (15.40)	5.85 (12.99)	15.00 (18.38)
**FQL pleasant, pre**	3.36 (9.42)	3.89 (8.65)	11.00 (18.20)	12.58 (19.24)	4.90 (9.60)	7.59 (12.88)
**FQL pleasant, post**	15.68 (23.19)	7.50 (14.80)	30.00 (28.84)	11.93 (18.07)	31.06 (26.96)	15.64 (21.67)

Unless otherwise indicated, data represent mean values and standard deviations

*ACT* perceived problems with activity, higher scores indicate more perceived problems with activity (range 3–21), *CBT* cognitive behavioral therapy, *FCS* fatigue catastrophizing scale, higher scores indicate more catastrophizing (range 10–50), *FQL* fatigue quality list, higher scores indicate a higher appraisal of fatigue as frustrating, exhausting, frightening, and pleasant (range per subscale 0–100), *IMQ* illness management questionnaire, subscale focusing on fatigue, higher scores indicate more focusing on fatigue (range 9–54), *pre* pre-assessment, *post* post-assessment, *RCT* randomized controlled trial, *SES* self-efficacy scale, higher scores indicate a higher sense of control over fatigue (range 7–28)

*As the 2 CBT groups (protocol-driven therapist feedback, on-demand therapist feedback) did not differ in their effect on fatigue, data were combined for the current analyses

### Outcome Variable

Fatigue severity was assessed with the fatigue severity subscale of the CIS-fatigue [37]. Eight items (e.g., “I feel tired”; see the Electronic Supplementary Material 2 for an overview of all items) assess fatigue severity over the 2 weeks prior to the assessment. Responses are scored on a 7-point Likert scale, ranging from (1) “Yes, that is true” to (7) “No, that is not true.” Higher scores indicate more severe fatigue (range 8–56). Previous research supports the reliability and validity of the CIS [38, 39].

### Potential Mechanism Variables

Fatigue catastrophizing was assessed with the 10-item fatigue catastrophizing scale (FCS; [40]), self-efficacy was assessed with the 7-item self-efficacy scale (SES; [41]), focusing on fatigue was assessed with the 9-item subscale focusing on symptoms of the illness management questionnaire (IMQ; [42]), problems with activity were assessed with the 3-item activity subscale of the CIS (ACT; [37]), and perceptions of fatigue were assessed with the 18-item fatigue quality list (FQL; [28], subscales: frustrating, exhausting, frightening, and pleasant). Previous research shows adequate psychometric properties of these scales [28, 38, 40, 42–44]. For detailed information of each measure, see Electronic Supplementary Material 3.

### Statistical Analyses

Oort’s SEM approach was applied for each of the three RCTs separately, with multi-group models to investigate change and possible response shift in both the CBT and control groups. For more details on the rationale of the SEM approach, the reader is referred to Oort [34]; an illustrative application is provided by Oort, Visser, and Sprangers [45].

**Fig. 2:**
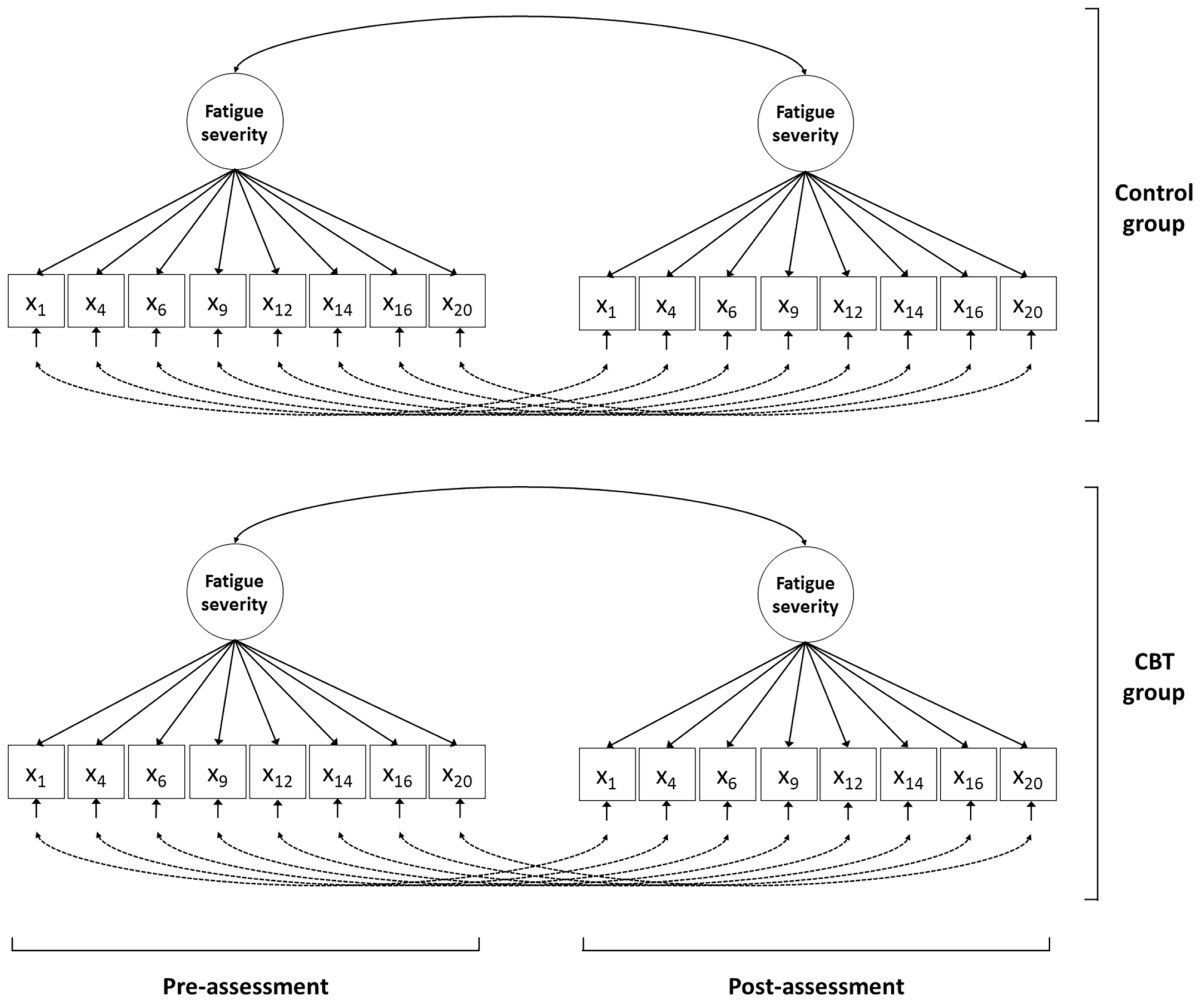
Multi-group longitudinal measurement model for fatigue severity. *Note.* The longitudinal measurement model of fatigue severity as measured with the CIS-fatigue is a one-factor model, where the underlying latent variable (i.e., fatigue severity) is measured by eight observed scores (i.e., the eight item scores of the subscale), both at pre- and post-assessment. The squares represent the observed item scores (*X*) measured at both pre- and post-assessment. The numbers 1 to 20 refer to the item numbers of the CIS-fatigue items (see Electronic Supplementary Material 2). The solid single-headed arrows at the bottom represent the residual factors of each item. The dotted double-headed arrow represents the longitudinal relations between the residual factors, where only the residual factors of the same item are allowed to correlate. The circles represent the construct that the items aim to measure (i.e., fatigue severity, both at pre- and post-assessment). Each arrow from a circle to an item represents a factor loading. The double-headed arrows between the circles represent the correlations between fatigue severity over time. The longitudinal measurement model is fitted in both the cognitive behavioral therapy (CBT) group and the control group simultaneously to enable the simultaneous assessment of change in fatigue severity and response shift

The approach is conducted in four steps. In step 1, the “measurement model” is established by specifying the relationships between the observed scores and the underlying latent variables, i.e., the target construct fatigue severity measured by eight questionnaire items at pre- and post-assessment in both CBT and control groups (see Fig. 2). The measurement model does not contain any constraints across groups or assessments. To achieve identification of all model parameters, scales and origins of the underlying latent variables were established by fixing the factor means at zero and the factor variances at one. The fit of the measurement model was evaluated using the chi-square (*χ*^2^) test of exact fit, and the root mean square error of approximation (RMSEA) as a measure of approximate fit. A non-significant chi-square test indicates exact model fit. An RMSEA value below 0.08 and below 0.05 indicate reasonable and close fit, respectively [46]. When the measurement model shows adequate fit, this indicates that the measurement pattern (i.e., the one-factor model of fatigue severity) is tenable in both groups and across both assessments.

In step 2, a “no response shift model” is specified in which all parameters associated with response shift (i.e., intercepts and factor loadings for the detection of recalibration and reprioritization/reconceptualization, respectively) are constrained to be equal across pre- and post-assessment. Identification of all model parameters is achieved by fixing only first occasion latent variable means and variances, as the latent variable means and variances of follow-up assessment are identified through the imposed equality restrictions. Its fit can be compared to the fit of the measurement model using the difference in chi-square test statistics (*Δχ*^2^). Significant deterioration in model fit indicates the presence of overall response shift (without distinguishing how response shift occurs). This omnibus test for response shift protects against false positives, whereas specific tests for specific indications of response shift effects have more statistical power. For example, the power to detect a difference in factor loading of Cohen’s *r* = 0.3 (i.e., a moderate sized change in factor loading) and a difference in intercept of Cohen’s *d* = 0.5 (i.e., a moderate sized change in intercept) would be 0.90, 0.42, and 0.34 with the omnibus test, but 1, 0.89, and 0.81 for the specific test in the CFS-trial, Cancer-trial, and Diabetes-trial, respectively (see Electronic Supplementary Material 4 for the power calculations). Due to the exploratory nature of this study, an increased false positives rate was deemed acceptable in favor of higher power to detect small but meaningful effects (defined in terms of clinical significance as indicated by the effect size value; see [47, 48]). Therefore, the investigation of response shift was also continued when the omnibus test for response shift was not significant. Note that there are no restrictions imposed across groups. That is, measurement equivalence is only investigated longitudinally (i.e., response shift), and not cross-sectionally (i.e., multi-group).

In step 3, it was investigated how each item is affected by response shift. Change in the pattern of factor loadings (i.e., a factor loading becoming zero) is indicative of reconceptualization, change in the value of factor loadings is indicative of reprioritization, and change in the intercepts is indicative of recalibration^1^ [34]. The detection of response shift was done in a step-by-step approach, where significant modification indices [49] were used to guide the identification of response shift and each modification was tested using the *Δχ*^2^ test statistic. In each step, it was also considered whether the detected effect was theoretically plausible, to ensure interpretability of the model. When a change in one of the model parameters was evidenced, it was investigated whether this effect was present in both CBT and control groups by testing the equality restriction on the specific model parameter in each group separately (using the *Δχ*^2^ test statistic). The final model including all indications of response shift is called “response shift model.” Identification restrictions in this model are the same as in the model for step 2.

In step 4, the impact of detected response shift on the intervention effect was investigated. First, the impact of response shift on the estimated change in the item(s) for which response shift was detected was calculated. Because change in the items is estimated as a function of model parameters, one can calculate the extent to which detected response shift effects (i.e., changes in intercepts and/or factor loadings) impact the estimated change in the associated items (for more information, see [50]). Second, the impact of response shift on the estimated change in the target construct fatigue severity was evaluated. Comparing the estimated change in fatigue severity between the response shift model and the no response shift model provides an indication of the impact of response shift on the overall intervention effect of CBT. Cohen’s *d* effect-size indices [47] were used for change in fatigue severity and impact of detected response shift on change in the items, where mean change was divided by the standard deviation of change. Means and standard deviations were derived from SEM parameters. Values of 0.3, 0.5, and 0.8 are indicative of small, moderate, and large effects, respectively [51]; effects of at least moderate size were considered to be clinically meaningful [48, 52].

The aim of the additional step 5 was to investigate whether change(s) in factor loading(s) and/or intercept(s) — as detected in step 3 — were associated with changes in a priori selected variables that would qualify as possible mechanisms for response shift consistent with the theoretical models of response shift [15, 16], see Fig. 1. A visual representation of the model used in step 5 is provided as Electronic Supplementary Material 5. Identification of the latent variables of fatigue severity was done in the same way as in step 2. The mechanism variables are included as single-indicator variables, where the latent variable is identified by restricting the (single) factor loading to one and the residual variance to zero. To aid interpretation, the change scores of the mechanism variables were calculated such that a negative score indicates improvement, that is, a decrease in the negative constructs (FCS, IMQ, ACT, FQL: frustrating, exhausting, frightening) or an increase in the positive constructs (SES, FQL: pleasant). This scoring is consistent with that of changes in fatigue severity (i.e., a negative score indicates improvement in fatigue). The change scores of the mechanism variables were modeled to correlate with the underlying latent variable fatigue severity (i.e., the target construct) at both occasions. This means that the mechanism variables are associated with the scores on the items of the fatigue severity questionnaire, through their association with fatigue severity as the target construct. A significant direct effect of a mechanism variable on an item thus indicates that there is a relation between change in the mechanism variable and change in the associated item that cannot be explained by changes in the target construct (i.e., fatigue severity). Such an effect evokes a process of change similar to that of response shift effects as detected in step 3 of the SEM approach (see also [53]). Based on response shift theory, a significant effect of a mechanism variable on an item affected by response shift is taken to indicate that change in the mechanism variable is a possible explanation for the detected response shift in that item. Because of the relative novelty of this approach to explore possible mechanisms of response shift, the results need to be interpreted in an exploratory fashion (however, see also [22, 54] for applications of the same procedure). The effects of mechanism variables can be represented as Cohen’s *r*, where values of 0.1, 0.3, and 0.5 are indicative of small, moderate, and large effects, respectively [51].

All statistical analyses were performed using Lavaan [55]. The maximum likelihood estimator with robust standard errors and mean- and variance-adjusted test statistics was used to take into account skewness of the data. Additional advantages of this procedure are that it performs well in terms of parameter estimation and protection against false positives (e.g., [56, 57]). RMSEA values were based on the adjusted test statistics. As the difference between two scaled goodness-of-fit test statistics does not yield the correct scaled difference test statistic, a scaled chi-square difference test [58] was applied. Syntax of the reported analyses is provided in the Electronic Supplementary Material 6.

## Results

### CFS-Trial

#### Objective 1: The occurrence of response shift

The multi-group longitudinal measurement model (see Fig. 2) with an added residual covariance between item 14 and item 20 showed the best fit (see Table 2). Adding the covariance seemed to make sense given the similarity of the wording of these items (see Electronic Supplementary Material 2). As expected, the no response shift model showed a significant deterioration in model fit as compared to the measurement model (*Δχ*^2^ (28) = 45.79, *p* = .018), indicating the overall presence of response shift. The equality restriction on the factor loading of item 4 (“Physically, I feel exhausted”) was found not to be tenable (*Δχ*^2^ (2) = 9.76, *p* = .007), indicating the presence of response shift through reprioritization. As expected, this response shift was only significant in the CBT group (*Δχ*^2^ (1) = 10.19, *p* = .001) and not in the control group (*Δχ*^2^ (1) = 0.53, *p* = .467). Inspection of parameter estimates showed that the item “Physically, I feel exhausted” became less important to the measurement of fatigue severity at follow-up, indicating that the observed decrease in this item was smaller than what would be expected if the item was still equally important to the measurement of fatigue severity.

**Table 2:** Overall goodness of fit of the models in steps 1–3 of the SEM approach for investigation of response shift (Objective 1)

	**Chi-square**	**Df**	***p*-value**	**RMSEA [90% CI]**
**CFS-trial**				
Step 1: Measurement model	266.30	190	< .001	0.060 [0.042–0.076]
+ residual covariance items 14–20	222.90	186	.033	0.042 [0.013–0.062]
Step 2: No response shift model	264.15	214	.011	0.046 [0.023–0.063]
Step 3: Response shift model	259.47	213	.016	0.044 [0.020–0.062]
**Cancer-trial**				
Step 1: Measurement model	250.11	190	.002	0.072 [0.045–0.095]
+ residual covariance items 14–20	236.15	186	.008	0.066 [0.036–0.090]
Step 2: No response shift model	271.33	214	.005	0.066 [0.038–0.089]
Step 3: Response shift model	264.64	213	.009	0.063 [0.033–0.086]
**Diabetes-trial**				
Step 1: Measurement model	217.06	190	.087	0.052 [0.000–0.082]
+ residual covariance items 14–20	206.61	186	.143	0.046 [0.000–0.078]
Step 2: No response shift model	244.73	214	.073	0.052 [0.000–0.080]
Step 3: Response shift model	241.92	213	.085	0.050 [0.000–0.079]

A non-significant chi-square test indicates exact model fit. A root mean square error of approximation (RMSEA) value below .08 indicates reasonable fit and a value below .05 indicates close fit [46]

*CI* confidence interval *Df* degrees of freedom

#### Objective 2: The impact of response shift on the intervention effect of CBT

The impact of detected response shift on the change in the item “Physically, I feel exhausted” was large, Cohen’s *d* = 0.93. However, the decrease in fatigue severity in the CBT group was of the same size with and without taking response shift into account (*d* =  − 1.12). Hence, other than expected, the detected response shift did not impact the intervention effect.

#### Objective 3: Cognitions and perceptions as mechanisms of response shift

There were no significant associations between the selected mechanism variables and the detected response shift in item 4. Hence, other than expected, the response shift effect was not associated with changes in cognitions and perceptions.

### Cancer-Trial

#### Objective 1: The occurrence of response shift

The multi-group longitudinal measurement model with an added residual covariance between item 14 and item 20, as in the CFS-trial, showed the best fit (see Table 2). The no response shift model did not show a statistically significant deterioration in model fit as compared to the measurement model (*Δχ*^2^ (28) = 35.89, *p* = .145). This indicates that there is no overall presence of response shift. To prevent missing small but meaningful effects, the investigation for possible specific indications of response shift was continued. The equality restriction on the factor loading of item 16 (“I tire easily”) was not tenable (*Δχ*^2^ (2) = 13.16, *p* = .001), indicating the presence of response shift through reprioritization. As expected, this response shift was only significant in the CBT group (*Δχ*^2^ (1) = 9.65, *p* = .002) and not in the control group (*Δχ*^2^ (1) = 1.51, *p* = .219). Inspection of parameter estimates showed that the item “I tire easily” became more important to the measurement of fatigue severity at follow-up, indicating that the observed decrease in this indicator was larger than what would be expected if the item was still equally important to the measurement of fatigue severity.

#### Objective 2: The impact of response shift on the intervention effect of CBT

The impact of detected response shift on change in the item “I tire easily” was large (*d* =  − 0.85). However, the decrease in fatigue severity in the CBT group was of comparable size with (*d* =  − 1.77) and without (*d* =  − 1.81) taking response shift into account. Hence, other than expected, the detected response shift did not impact the intervention effect.

#### Objective 3: Cognitions and perceptions as mechanisms of response shift

The FQL subscales exhausting and frightening and the FCS showed small but significant associations with item 16 (*r* =  − 0.24, *p* < .001; *r* = .14, *p* = 0.041; *r* = 0.20, *p* = .035, respectively). Thus, as expected, it seemed that the detected response shift in item 16 was associated with changes in cognitions and perceptions. Specifically, an evaluation of fatigue being less exhausting was less strongly associated to improvement on the item “I tire easily” as compared to the other items of fatigue severity. An evaluation of fatigue as less frightening and a reduction in fatigue catastrophizing were more strongly associated with improvement on item 16 than with the other items.

### Diabetes-Trial

#### Objective 1: The occurrence of response shift

The multi-group longitudinal measurement model with, again, an added residual covariance between item 14 and item 20 showed the best fit (see Table 2). The no response shift model did not show a statistically significant deterioration in model fit as compared to the measurement model (*Δχ*^2^ (28) = 40.86, *p* = .055). This indicates that there is no overall presence of response shift. Nevertheless, the investigation for possible specific indications of response shift was continued. Inspection of modification indices showed response shift through reprioritization in item 16 (“I tire easily”; *Δχ*^2^ (2) = 7.30, *p* = .026). As expected, this response shift was only significant in the CBT group (*Δχ*^2^ (1) = 7.39, *p* = .007) and not in the control group (*Δχ*^2^ (1) = 0.78, *p* = .376). Inspection of parameter estimates showed that, similar to the results of the Cancer-trial, the item “I tire easily” became more important to the measurement of fatigue severity at follow-up.

#### Objective 2: The impact of response shift on the intervention effect of CBT

The impact of detected response shift on change in the item “I tire easily” was large (*d* =  − 0.89). However, the decrease in fatigue severity in the CBT group was of comparable size with (*d* =  − 2.02) and without (*d* =  − 2.05) taking response shift into account. Hence, other than expected, the detected response shift did not impact the intervention effect.

#### Objective 3: Cognitions and perceptions as mechanisms of response shift

The FQL subscale pleasant and the SES showed small but significant associations with item 16 (*r* = 0.26, *p* = .009; *r* =  − 0.20, *p* = .024, respectively). Thus, as expected, some indication of associations between changes in cognitions and perceptions and the detected response shift was found. Perceiving fatigue as more pleasant was more strongly related to improvement on the scores of the item “I tire easily,” whereas improvement in self-efficacy was less strongly related to improvement on the scores of this item, as compared to the associations with the other items of fatigue severity.

## Discussion

The presented results suggest that response shift occurred in the measurement of fatigue in patients undergoing CBT targeting severe fatigue. Response shift through reprioritization occurred in two of the eight items assessing fatigue, that is, “Physically, I feel exhausted” (CFS-trial) and “I tire easily” (Cancer- and Diabetes-trials). In all three trials, as expected, indications for response shift were found only in the CBT group and not in the control group, suggesting that indeed CBT acts as a catalyst inducing a change in patients’ interpretation of their fatigue. Yet, the occurrence of response shift did not have an impact on the estimated intervention effect, suggesting that the measured reduction in fatigue after CBT reflects an improvement in patients’ fatigue, not its re-evaluation. Changes in some fatigue-related cognitions and perceptions showed small associations with the detected response shift in the item “I tire easily.”

After CBT, the item “Physically, I feel exhausted” became less important to the measurement of fatigue in the CBT group of the CFS-trial, indicating that scores on this item dropped *less* relative to the other items of the CIS-fatigue. In terms of response shift, this may indicate that patients report less decline in physical exhaustion because they have re-interpreted it as a more normal consequence of daily activity and hence as being less indicative of their chronic fatigue. This would be in line with one of the aims of CBT, namely the normalization of the experience of being fatigued. In the Cancer- and Diabetes-trials, the item “I tire easily” became more important to the measurement of fatigue in both CBT groups, indicating that scores on this item dropped *more* relative to the other items of the CIS-fatigue. This may indicate that the meaning of this item changed, such that the perception of tiring easily became of more relevance for patients’ fatigue. No indications for the occurrence of recalibration and reconceptualization were found. As described in the qualitative literature [59, 60], individuals suffering from severe and chronic fatigue describe their symptom as more intense and of a different nature than any fatigue experienced before. It might be that this initial experience with being severely fatigued leads individuals to develop a new standard (recalibration) and meaning (reconceptualization) of fatigue, which is then not easily changed (i.e., re-evaluated) through CBT. In other words, recalibration and reconceptualization may already have occurred prior to CBT.

Unexpectedly, taking the occurrence of response shift into account had no impact on the estimated intervention effect. Put differently, while CBT induced a discrepancy between the observed scores and target construct, this did not affect the measured reduction in fatigue severity after CBT. This is in line with recent studies that found a limited impact of response shift on change in the target construct [22, 54]. While the detected response shifts in this study were of a large magnitude, they occurred only on a single item per trial, thereby limiting their impact on overall fatigue severity, as measured by eight items. It could be that no impact on fatigue severity was found due to insufficient sensitivity of the analytical method to detect response shift, or possible heterogeneity of response shift. For example, it might be that some participants experience larger response shift, whereas others experience no or very little response shift, which goes undetected when investigating response shift in the entire group. Future research is needed to investigate whether response shift occurs and impacts overall fatigue severity in specific subgroups. For example, it might be that patients with a relative short duration of severe fatigue are more inclined to (re-)evaluate fatigue than patients who have experienced it for many years.

In the Cancer- and Diabetes-trials, changes in some fatigue-related cognitions and perceptions showed small associations with the detected response shift on the item “I tire easily.” Measures of changes in cognitions and perceptions might not be perfect operationalizations of changes in meaning and the method applied to investigate their relation is not well established yet (however, see also [22, 54]). Nevertheless, these results may suggest that cognitions and perceptions could be part of the mechanisms underlying the detected response shift.

A strength of this study is the assessment of response shift in three RCTs in which individuals with different chronic conditions underwent the same CBT for severe fatigue. To the best of our knowledge, this is the first study that investigates response shift in fatigue in the context of CBT. The results of the three trials combined suggest that CBT has a comparable effect on fatigue and its measurement across different patient groups. Further, while items in which response shift occurred differed between trials, explorative analyses showed that parameter changes in the CFS-trial were in the same direction for the item “I tire easily,” and parameter changes in the Cancer- and Diabetes-trials were in the same direction for the item “Physically, I feel exhausted” (but not significantly; data not shown).

While the consistency of the results is encouraging, limitations regarding the statistical analyses and theoretical assumptions need to be considered: First, the analyses were conducted in an exploratory fashion. In the Cancer- and Diabetes-trials, the investigations of response shift were continued despite non-significant omnibus tests. In this first, exploratory phase of response shift research following CBT, this approach was chosen to not miss statistically small but potentially meaningful effects. That is, given the sample size of especially the Cancer- and Diabetes-trials, the power to detect effects with the omnibus test was low as compared to the power to detect effects with a test on a specific response shift. To prevent losing power for the test of individual effects, no correction for multiple testing was used. As a consequence, the risk of detecting false positives (i.e., making type I errors) may have increased. However, the chosen statistical procedure performs well with regard to type I error and in terms of finding correct estimates of factor loadings [56, 57]. Nevertheless, given the insignificant omnibus test, future studies are needed to provide insight into the stability of the current findings.

Another limitation is that no across group measurement invariance restrictions were imposed, making direct comparison of the latent variables across groups unwarranted. That is, when the measurement structure of fatigue severity is not invariant across groups, differences in the means of the underlying latent variables are not necessarily indicative of differences in fatigue severity. Measurement equivalence was evaluated across groups at baseline (results not shown) which confirmed successful randomization in all trials. It was not the aim to directly compare fatigue severity between groups, but rather to investigate whether there would be changes in the measurement structure of fatigue over time (i.e., response shift) in either or both groups. By focusing the analyses on response shift investigation only, possible response shift effects would not be obscured by across group differences. Future research is needed to investigate whether there are informative differential effects between groups.

The validity of the SEM approach for detection of response shift depends on the extent to which certain methodological and theoretical assumptions hold. First, application of SEM requires that response shift occurs in the majority of the sample and in a minority of the indicators. Group-level results may not be directly meaningful for inferences about individual-level processes (e.g., some individuals may show no response shift or even response shift in the opposite direction). Nevertheless, results from the SEM approach can be meaningful for the interpretation of general patterns of response shift, similar to general patterns of treatment effectiveness as measured by group-level change in fatigue severity.

Second, the detected measurement noninvariance is taken to indicate response shift. This interpretation is only valid when the detected effects are indeed caused by a change in meaning of the subjective evaluations [15]. With SEM, the interpretations of the different types of noninvariance are consistent with the different pathways through which response shift can occur (i.e., recalibration, reprioritization, and reconceptualization). Theoretical, clinical, and common sense arguments were used to substantiate the interpretation of results as response shift. Still, there may be other explanations for the detected effects. For example, the finding that the item “Physically, I feel exhausted” became *less* and “I tire easily” became *more* important, respectively, to the measurement of fatigue could also indicate that CBT is relatively less vs. more effective regarding these aspects of fatigue. Qualitative research can help to investigate respondents’ interpretation of these scores [61]. To further substantiate the interpretation of findings as indications of response shift, the associations between detected response shift effects and potential mechanism variables, according to response shift theory [36, 62], were also investigated. Moreover, it seems likely that CBT can induce response shift as it directly targets patients’ cognitions and perceptions regarding their fatigue: patients are challenged to change maladaptive cognitions about fatigue (e.g., “the fatigue forces me to become inactive”) into adaptive ones (e.g., “despite being fatigued I can gradually increase my level of activity”) and learn to (re-)evaluate fatigue as a normal everyday experience (e.g., “it is normal to become fatigued after a late night, and I will recover from it”). Such change in fatigue-related beliefs could lead to a change in response to the self-report questionnaire, despite an equal level of fatigue. The findings from a study on treatment of depression are in line with this assumption. The authors found (indicators of) response shift to be more pronounced in individuals who received psychotherapy, including CBT, as compared to those who received medication [23].

Lastly, a total of 37 individuals were excluded from analyses. Data of 29 individuals were excluded to ensure that the groups clearly differed in their exposure to a catalyst (here: CBT). Data from eight individuals were excluded due to attrition and consequent missing data at post-assessment. As the number of individuals excluded due to missing data was small (< 2% of the total data), it is unlikely that this would have impacted the results.

In sum, across patient groups, CBT seems to have an impact on the measurement of fatigue that is indicative of response shift. However, these effects have not been shown to bias the estimated effect of CBT on fatigue severity. Some of the cognitions and perceptions targeted during CBT are associated with the detected response shift, making them possible mechanism variables that warrant further investigation. Future qualitative research is needed to examine the assumption that CBT acts as a catalyst that induces a change in patients’ interpretation of different aspects of fatigue.

## Supplementary Information

Below is the link to the electronic supplementary material.

Supplementary file1 (PDF 558 KB)

## Footnotes

1 Note that change in intercept values is also referred to as uniform recalibration, where nonuniform recalibration refers to a change in residual variances. As the latter is not relevant for the investigation of mean change [34], we here only consider change in intercept values and refer to this as recalibration for reasons of conciseness.
