# Supplementary material for: Response Shift After Cognitive Behavioral Therapy Targeting Severe Fatigue: Explorative Analysis of Three Randomized Controlled Trials
Source: Int J Behav Med. 2022 Jul 22;30(4):473–85. doi: 10.1007/s12529-022-10111-8 (PMC10310616; doi:10.1007/s12529-022-10111-8)
Supplement: Supplementary file 1 — Supplementary file1 (PDF 558 KB) [file 12529_2022_10111_MOESM1_ESM.pdf]

Electronic Supplementary Material 1: Description of the three randomized controlled trials

CFS-trial, Janse [1,2]

Individuals meeting the chronic fatigue syndrome (CFS) diagnosis according to the Centers for Disease Control (CDC) consensus criteria revised in 2003 and referred to the Expert Center for Chronic Fatigue, a Dutch tertiary treatment facility for chronic fatigue, were recruited and considered eligible for participation if they were (1) severely fatigued as indicated by a score of ≥ 35 on the fatigue subscale of the Checklist Individual Strength (CIS-fatigue), (2) severely disabled as indicted by a score of ≥ 700 on the Sickness Impact Profile, (3) aged ≥ 18 years, (4) able to speak, read, and write Dutch, and (5) able to use a computer and having access to the internet. Patients were excluded from participation if they were (1) involved in legal procedures concerning disability benefit claims or (2) participating in other CFS research.

In this 3-arm randomized controlled trial (RCT), patients were randomly allocated to internet-based CBT with protocol-driven therapist feedback, internet-based CBT with on-demand therapist feedback, or a waitlist. Assessments were performed before randomization (pre-assessment) and after the intervention or waiting period (post-assessment), that is, 6 months later.

CBT group

Internet-based CBT was provided on a secured webportal under guidance of a therapist. Patients received seven modules, starting with a module on formulating treatment goals, followed by the modules ‘Regulate sleep-wake cycle’, ‘Helpful beliefs about fatigue’, ‘How to communicate with others about CFS’, ‘Gradually increasing my activities’ and ‘Reaching my goals step by step’. CBT was concluded with a final evaluation module. The treatment was tailored to patients’ activity pattern as assessed with actigraphy.

In the protocol-driven feedback condition, patients were asked to report on their progress by e-mail according to a prescribed schedule. The therapist provided feedback and sent reminders if the schedule was not adhered to. In the feedback-on-demand condition, feedback was only provided when the patient indicated a need for advice. As the two CBT groups did not significantly differ in their effect on fatigue, these groups were combined for the analyses reported in the paper.

Control group

After the post-assessment, patients from the waitlist were offered face-to-face CBT.

Cancer-trial, Abrahams [3,4]

Individuals who had breast were recruited from Dutch hospitals and via self-referral and were considered eligible for participation if they (1) were treated with curative intent, (2) had completed cancer treatment since ≥ 3 months (barring hormone and targeted therapy) and (3) had no evidence of disease recurrence. Further inclusion criteria were being (4) female, (5) aged ≥ 18 years, (6) severely fatigued as indicated by a score of ≥ 35 on the CIS-fatigue, (7) able to speak, read, and write Dutch, and (8) able to use the internet. Patients were excluded from participation if they (1) had a comorbidity that could explain their severe fatigue, (2) had a depressive disorder (assessed with the Beck Depression Inventory for Primary Care and, in case of a score of ≥ 4, the depression module of the Mini- International Neuropsychiatric Interview), (3) were undergoing current psychological treatment for a psychiatric disorder or (4) were currently undergoing CBT for fatigue.

Patients were randomized to internet-based CBT or a waitlist, stratified by time since completion of cancer treatment (3-12 months vs. ≥ 12 months) and type of referral (hospital vs. self-referral). Assessments were performed before randomization (pre-assessment) and after the intervention or waiting period (post-assessment), that is, 6 months later.

CBT group

Patients initiated their treatment with two face-to-face sessions, after which they followed their treatment online on a secure webportal under guidance of their therapist. All patients started with a module on formulating treatment goals, followed by up to six modules; these were: 'Sleep-wake rhythm', 'Helpful thinking', 'Activity regulation', 'Social Support' and the two cancer-specific modules 'Coping with cancer and cancer treatment' and 'Fear of cancer recurrence'. CBT was concluded with a final evaluation module. Patients followed modules that were indicated for them based on scores on relevant questionnaires assessed at baseline and tailored to their activity pattern.

Control group

After the post-assessment, patients from the waitlist were offered face-to-face CBT.

Diabetes-trial, Menting [5,6]

Individuals with type I diabetes were recruited from Dutch hospitals and via self-referral and were considered eligible for participation if they (1) had been diagnosed with type 1 diabetes for ≥ 1 year, (2) were aged 18–70 years, (3) were able to speak, read, and write Dutch, (4) were severely fatigued as indicated by a score of ≥ 35 on the CIS-fatigue, (5) reported a duration of fatigue of ≥ 6 months. Patients were excluded from participation if they (1) had moderate-to-severe renal failure, (2) were blind or had severe visual impairment, (3) had a medical history of congestive heart failure, (4) had a medical history of a stroke in the past 5 years, (5) had a BMI of ≥ 40 kg/m^2, (6) were wheelchair dependent or (7) had a concurrent psychiatric or medical comorbidity that could explain their fatigue.

Patients were randomized to blended CBT or a waitlist, stratified by type of referral (hospital vs. self-referrals). Assessments were performed before randomization (pre-assessment) and after the intervention or waiting period (post-assessment), that is, 5 months later.

CBT group

CBT was provided in blended form consisting of five to eight face-to-face sessions and web-based modules offered on a secured webportal. All patients started with a module on formulating treatment goals, followed by up to six modules; these were: ‘Regulation of the sleep-wake pattern’, ‘Formulating helpful fatigue-related beliefs’, ‘Activity regulation and increasing the level of activity’, ‘Optimalisation of social support and interactions’ and the two diabetes-specific modules ‘Coping with pain’ and ‘Reducing diabetes-related distress’. CBT was concluded with a final evaluation module. Patients followed modules that were indicated for them based on scores on relevant questionnaires assessed at baseline and tailored to their activity pattern.

Control group

After the post-assessment, patients from the waitlist were offered blended CBT.

Electronic Supplementary Material 2: Items of the Checklist Individual Strength, subscale fatigue severity

Item number	Item wording	Response categories		
Item 1	I feel tired*	Yes, that is true	7 ----- 1	No, that is not true
Item 4	Physically, I feel exhausted*	Yes, that is true	7 ----- 1	No, that is not true
Item 6	I feel fit	Yes, that is true	1 ----- 7	No, that is not true
Item 9	I feel weak*	Yes, that is true	7 ----- 1	No, that is not true
Item 12	I feel rested	Yes, that is true	1 ----- 7	No, that is not true
Item 14	Physically I am in bad shape*	Yes, that is true	7 ----- 1	No, that is not true
Item 16	I tire easily*	Yes, that is true	7 ----- 1	No, that is not true
Item 20	Physically I feel I am in good shape	Yes, that is true	1 ----- 7	No, that is not true

Note. * indicates reversed scoring. Items are scored in a way that higher scores indicate more fatigue.

Electronic Supplementary Material 3: Detailed description of instruments to assess potential mechanism variables

Fatigue catastrophizing was assessed with the fatigue catastrophizing scale (FCS, [7]). Ten items (e.g., “I imagine the fatigue becoming even more intense and exhausting”) assess patients’ negative cognitions and feelings of helplessness about fatigue, referring to situations when feeling fatigued. Responses are scored on a 5-point Likert scale from (1) “Never true” to (5) “All of the time true”. Higher scores indicate more catastrophizing (range 10-50).

Self-efficacy was assessed with the self-efficacy scale (SES, [8]). Seven items (e.g., “I think I could positively influence my fatigue”) assess patients’ perceived control over their fatigue. Responses are scored on a 4-point scale ranging from (1) “No, I am convinced that is not true” to (4) “Yes, I am convinced that is true”. Higher scores indicate a higher sense of control over fatigue (range 7-28).

Focusing on fatigue was assessed with the subscale focusing on symptoms of the illness management questionnaire (IMQ, [9]). Nine items (e.g., “I spend a lot of time thinking about my fatigue”) assess patients’ focus on fatigue during the previous month. Responses are scored on a 6-point Likert scale, ranging from (1) “Never” to (6) “Always”. Higher scores indicate more focusing on fatigue (range 9-54).

Problems with activity were assessed with the activity subscale of the CIS (ACT, [10]). Three items (e.g., “I think I do very little in a day”) assess patients’ perception of their daily activity over the 2 weeks prior to the assessment. Responses are scored on a 7-point Likert scale (see paper). Higher scores indicate more perceived problems with activity (range 3-21).

Perceptions of fatigue were assessed with the fatigue quality list (FQL, [11]). The questionnaire consists of eighteen adjectives describing fatigue (e.g., “upsetting”, “exhausting”, “relaxing”). Patients are instructed to mark all adjectives that fit their experience of fatigue during the last 2 weeks. Four subscales (frustrating, exhausting, frightening and pleasant) are

calculated by summing the respective items. Higher scores indicate a higher appraisal of fatigue as frustrating, exhausting, frightening and pleasant (range per subscale 0-100).

Electronic Supplementary Material 4: Power analyses, conducted in R

```
#### SEM POWER

#### CIS-FATIGUE
#### Calculations for chi-based power overall test on response shift, i.e. what is the power to reject H0 (no response shift)?
#### H0: Model without response shift
#### H1: Model with response shift

#### Specification choices:
#### -> Response shift is only present in the cbt-group
#### -> We include one difference in intercepts (recalibration) and one difference in factor loadings (reprioritization)
####    -> Size of difference in intercept following Cohen's d = .5 (medium)
####    -> Size of difference in factor lading following Cohen's r = .3 (medium)
#### -> Other parameter estimates are specified as follows:
####    -> Factor loadings are .5 (large)
####    -> Factor variances are 1 (so that solution is standardized; aids interpretation and specification)
####    -> Factor correlation is .5
####    -> Factor means at post-assessment are .8 (cbt; large change) and .2 (control; small change)
####    -> Residual variances are 1-lambda^2 (so that variable variances are 1; solution is standardized)
####    -> Residual covariances are .2 (small-medium)
####    -> Intercept values are 1


#### MODEL H1 CBT GROUP: Response shift Model: Model including difference in intercept and factor loading over time

CIS.H1.TL.medium = '

	# factor loadings
	CISv =~ .5*cis1v + .5*cis4v + .5*cis6v + .5*cis9v + .5*cis12v + .5*cis14v + .5*cis16v + .5*cis20v
	CISn =~ .5*cis1n + .8*cis4n # different loading
		+ .5*cis6n + .5*cis9n + .5*cis12n + .5*cis14n + .5*cis16n + .5*cis20n

	# (co)variances common factor
	CISv ~~ 1*CISv + .5*CISn
	CISn ~~ 1*CISn

	# residual (co)variances
	cis1v ~~ .75*cis1v + .2*cis1n
	cis4v ~~ .75*cis4v + .2*cis4n
	cis6v ~~ .75*cis6v + .2*cis6n
	cis9v ~~ .75*cis9v + .2*cis9n
	cis12v ~~ .75*cis12v + .2*cis12n
	cis14v ~~ .75*cis14v + .2*cis14n
	cis16v ~~ .75*cis16v + .2*cis16n
	cis20v ~~ .75*cis20v + .2*cis20n
```

```
	cis1n ~~ .75*cis1n
	cis4n ~~ .36*cis4n # different residual variance
	cis6n ~~ .75*cis6n
	cis9n ~~ .75*cis9n
	cis12n ~~ .75*cis12n
	cis14n ~~ .75*cis14n
	cis16n ~~ .75*cis16n
	cis20n ~~ .75*cis20n

	# model modification to require good fit
	cis14v ~~ .1*cis20v
	cis14n ~~ .1*cis20n

	# intercept values
	cis1v ~ 1.5*1 # difference intercept
	cis4v ~ 1*1
	cis6v ~ 1*1
	cis9v ~ 1*1
	cis12v ~ 1*1
	cis14v ~ 1*1
	cis16v ~ 1*1
	cis20v ~ 1*1

	cis1n ~ 1*1
	cis4n ~ 1*1
	cis6n ~ 1*1
	cis9n ~ 1*1
	cis12n ~ 1*1
	cis14n ~ 1*1
	cis16n ~ 1*1
	cis20n ~ 1*1

	# common factor means
	CISv ~ 0*1
	CISn ~ 0.8*1
'

#### MODEL H1 CONTROL GROUP: NO DIFFERENCES ACROSS TIME

CIS.H1.control = '

	# factor loadings
	CISv =~ .5*cis1v + .5*cis4v + .5*cis6v + .5*cis9v + .5*cis12v + .5*cis14v + .5*cis16v +
.5*cis20v
	CISn =~ .5*cis1n + .5*cis4n + .5*cis6n + .5*cis9n + .5*cis12n + .5*cis14n + .5*cis16n
+ .5*cis20n

	# (co)variances common factor
	CISv ~~ 1*CISv + .5*CISn
	CISn ~~ 1*CISn

	# residual (co)variances
	cis1v ~~ .75*cis1v + .2*cis1n
```

```
	cis4v ~~ .75*cis4v + .2*cis4n
	cis6v ~~ .75*cis6v + .2*cis6n
	cis9v ~~ .75*cis9v + .2*cis9n
	cis12v ~~ .75*cis12v + .2*cis12n
	cis14v ~~ .75*cis14v + .2*cis14n
	cis16v ~~ .75*cis16v + .2*cis16n
	cis20v ~~ .75*cis20v + .2*cis20n

	cis1n ~~ .75*cis1n
	cis4n ~~ .75*cis4n
	cis6n ~~ .75*cis6n
	cis9n ~~ .75*cis9n
	cis12n ~~ .75*cis12n
	cis14n ~~ .75*cis14n
	cis16n ~~ .75*cis16n
	cis20n ~~ .75*cis20n

	# model modification to require good fit
	cis14v ~~ .1*cis20v
	cis14n ~~ .1*cis20n

	# intercept values
	cis1v ~ 1*1
	cis4v ~ 1*1
	cis6v ~ 1*1
	cis9v ~ 1*1
	cis12v ~ 1*1
	cis14v ~ 1*1
	cis16v ~ 1*1
	cis20v ~ 1*1

	cis1n ~ 1*1
	cis4n ~ 1*1
	cis6n ~ 1*1
	cis9n ~ 1*1
	cis12n ~ 1*1
	cis14n ~ 1*1
	cis16n ~ 1*1
	cis20n ~ 1*1

	# common factor means
	CISv ~ 0*1
	CISn ~ 0.2*1
'


#### H0 multigroup: Model without response shift (all intercepts and factor loadings equal over time)

CIS.H0.MG = '

	# equal factor loadings across time (not groups)
	CISv =~		c(L1.con,L1.cbt)*cis1v +
				c(L4.con,L4.cbt)*cis4v +
```

```
                c(L6.con,L6.cbt)*cis6v +
                c(L9.con,L9.cbt)*cis9v +
                c(L12.con,L12.cbt)*cis12v +
                c(L14.con,L14.cbt)*cis14v +
                c(L16.con,L16.cbt)*cis16v +
                c(L20.con,L20.cbt)*cis20v
CISn =~         c(L1.con,L1.cbt)*cis1n +
                c(L4.con,L4.cbt)*cis4n +
                c(L6.con,L6.cbt)*cis6n +
                c(L9.con,L9.cbt)*cis9n +
                c(L12.con,L12.cbt)*cis12n +
                c(L14.con,L14.cbt)*cis14n +
                c(L16.con,L16.cbt)*cis16n +
                c(L20.con,L20.cbt)*cis20n

# (co)variances common factor
CISv ~~ 1*CISv + CISn
CISn ~~ CISn

# residual (co)variances
cis1v ~~ cis1v + cis1n
cis4v ~~ cis4v + cis4n
cis6v ~~ cis6v + cis6n
cis9v ~~ cis9v + cis9n
cis12v ~~ cis12v + cis12n
cis14v ~~ cis14v + cis14n
cis16v ~~ cis16v + cis16n
cis20v ~~ cis20v + cis20n

cis1n ~~ cis1n
cis4n ~~ cis4n
cis6n ~~ cis6n
cis9n ~~ cis9n
cis12n ~~ cis12n
cis14n ~~ cis14n
cis16n ~~ cis16n
cis20n ~~ cis20n

cis14v ~~ cis20v
cis14n ~~ cis20n

# equal intercepts across time (not groups)
cis1v ~ c(int1.con,int1.cbt)*1
cis4v ~ c(int4.con,int4.cbt)*1
cis6v ~ c(int6.con,int6.cbt)*1
cis9v ~ c(int9.con,int9.cbt)*1
cis12v ~ c(int12.con,int12.cbt)*1
cis14v ~ c(int14.con,int14.cbt)*1
cis16v ~ c(int16.con,int16.cbt)*1
cis20v ~ c(int20.con,int20.cbt)*1

cis1n ~ c(int1.con,int1.cbt)*1
cis4n ~ c(int4.con,int4.cbt)*1
cis6n ~ c(int6.con,int6.cbt)*1
```

```
	cis9n ~ c(int9.con,int9.cbt)*1
	cis12n ~ c(int12.con,int12.cbt)*1
	cis14n ~ c(int14.con,int14.cbt)*1
	cis16n ~ c(int16.con,int16.cbt)*1
	cis20n ~ c(int20.con,int20.cbt)*1

	# common factor means
	CISv ~ 0*1
	CISn ~ 1
'

#### use package lavaan for calculations
require(lavaan)

#### Step 1: Calculate population values under H1
cov1 <- fitted(lavaan(CIS.H1.control))$cov		# population values control group
means1 <- fitted(lavaan(CIS.H1.control))$mean
cov2 <- fitted(lavaan(CIS.H1.TL.medium))$cov		# population values cbt group
means2 <- fitted(lavaan(CIS.H1.TL.medium))$mean

#### Step 2: Fit H0 on H1 values to require ncp
CFS_ncp <-
fitMeasures(lavaan(CIS.H0.MG,sample.cov=list(cov1,cov2),sample.mean=list(means1,mean
s2),sample.nobs=list(73,149)),"chisq") # CFS-trial
Cancer_ncp <-
fitMeasures(lavaan(CIS.H0.MG,sample.cov=list(cov1,cov2),sample.mean=list(means1,mean
s2),sample.nobs=list(63,60)),"chisq") # Cancer-trial
Diabetes_ncp <-
fitMeasures(lavaan(CIS.H0.MG,sample.cov=list(cov1,cov2),sample.mean=list(means1,mean
s2),sample.nobs=list(58,49)),"chisq") # Diabetes-trial

#### Step 3: use ncp to compute power for overall test (df=28)
-> ncp: 31.454/12.736/10.423
crit.df28=qchisq(.05,28,lower.tail=FALSE)
1-pchisq(crit.df28,	28,	CFS_ncp)
1-pchisq(crit.df28,	28,	Cancer_ncp)
1-pchisq(crit.df28,	28,	Diabetes_ncp)

-> power overall: 0.922 / 0.445 / 0.356

#### Step 3: use ncp to compute power for specific test (df=2)
crit.df2=qchisq(.05,2,lower.tail=FALSE)
1-pchisq(crit.df2,	2,	CFS_ncp)
1-pchisq(crit.df2,	2,	Cancer_ncp)
1-pchisq(crit.df2,	2,	Diabetes_ncp)

-> power specific: 1 / 0.902 / 0.832
```

Electronic Supplementary Material 5: Visual representation of step 5 of the SEM approach to investigate possible mechanisms for detected response shift.

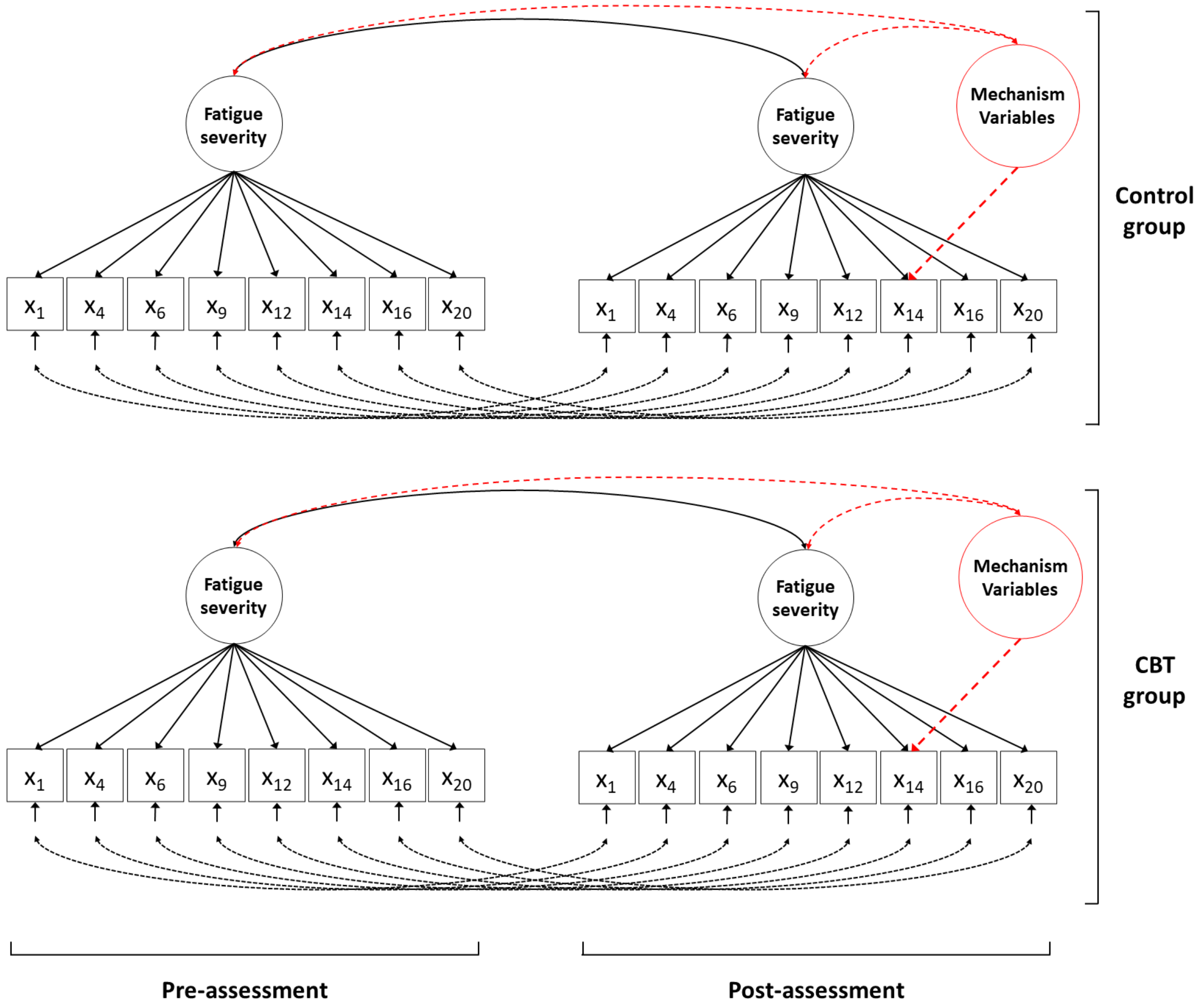

Note. In step 5 of the SEM approach the longitudinal factor model of fatigue severity (see Figure 2 for a detailed explanation of the part of the model represented with black lines) is extended to include possible 'Mechanism Variables' and their association(s) with detected response shift. The red circles represent all possible mechanism variables included in the model, i.e. changes in fatigue- and activity-related cognitions and perceptions. The dotted red arrow from 'Mechanism variables' to the item X_{14} represents a factor loading. The factor loading is only included in the model for the item(s) for which response shift was detected in step 3 of the SEM approach. The red dotted double-headed arrows between the circles represent the correlations between the mechanism variables and fatigue severity at both occasions. The model with possible mechanism variables included in the model is fitted in both the cognitive behavioral therapy (CBT) group and the control group simultaneously.

Electronic Supplementary Material 6: Syntax of analyses, conducted in R with lavaan;

exemplary for the CFS-trial

```
########################################
#### SEM ANALYSES CFS-TRIAL
########################################

# load required packages
require(foreign)        # for reading spss files
require(lavaan)         # for sem analyses

################
# DATA

# read spss file
[DATA] = read.spss("[NAME DATA FILE]", to.data.frame=TRUE, use.value.labels = FALSE)

# CIS subscale: Subjective feeling of fatigue
# Items 1, 4, 6, 9, 12, 14, 16, 20
# 7-point scale

################
# ANALYSES

# 1-factor model: unidimensional factor model of fatigue (7 CIS-items)
# multigroup: waitlist vs iCBT

## STEP 1: MEASUREMENT MODEL

CIS.1 = '

        # factorloadings
        CISpre =~ cis1v + cis4v + cis6v + cis9v + cis12v + cis14v + cis16v + cis20v
        CISpost =~ cis1n + cis4n + cis6n + cis9n + cis12n + cis14n + cis16n + cis20n

        # (co)variances common factor
        CISpre ~~ 1*CISpre + CISpost
        CISpost ~~ 1*CISpost

        # residual (co)variances
        cis1v ~~ cis1v + cis1n
        cis4v ~~ cis4v + cis4n
        cis6v ~~ cis6v + cis6n
        cis9v ~~ cis9v + cis9n
        cis12v ~~ cis12v + cis12n
        cis14v ~~ cis14v + cis14n
        cis16v ~~ cis16v + cis16n
        cis20v ~~ cis20v + cis20n

        cis1n ~~ cis1n
        cis4n ~~ cis4n
        cis6n ~~ cis6n
```

```
        cis9n ~~ cis9n
        cis12n ~~ cis12n
        cis14n ~~ cis14n
        cis16n ~~ cis16n
        cis20n ~~ cis20n

        # model modification to require good fit
        cis14v ~~ cis20v
        cis14n ~~ cis20n

        # intercept values
        cis1v ~ 1
        cis4v ~ 1
        cis6v ~ 1
        cis9v ~ 1
        cis12v ~ 1
        cis14v ~ 1
        cis16v ~ 1
        cis20v ~ 1

        cis1n ~ 1
        cis4n ~ 1
        cis6n ~ 1
        cis9n ~ 1
        cis12n ~ 1
        cis14n ~ 1
        cis16n ~ 1
        cis20n ~ 1

        # common factor means
        CISpre ~ 0*1
        CISpost ~ 0*1
'

# run multigroup model with Mean and Variance adjusted ML
CIS.1OUT <- lavaan( CIS.1,
                        data=[DATA],
                        group="[GROUPING VARIABLE]",
                        estimator="MLMV")

# output
summary(    CIS.1OUT,
            fit.measures=TRUE,
            standardized=TRUE)



## STEP 2: OVERALL TEST OF RESPONSE SHIFT

CIS.2 = '

        # equal factorloadings across time (not groups)
        CISpre =~     c(L1.con,L1.cbt)*cis1v +
                      c(L4.con,L4.cbt)*cis4v +
```

```
                      c(L6.con,L6.cbt)*cis6v +
                      c(L9.con,L9.cbt)*cis9v +
                      c(L12.con,L12.cbt)*cis12v +
                      c(L14.con,L14.cbt)*cis14v +
                      c(L16.con,L16.cbt)*cis16v +
                      c(L20.con,L20.cbt)*cis20v
        CISpost =~    c(L1.con,L1.cbt)*cis1n +
#                     c(L4.con,L4.cbt)*cis4n +
                      c(L4.con,L4n.cbt)*cis4n + ## unequal factorloading treatment group
                      c(L6.con,L6.cbt)*cis6n +
                      c(L9.con,L9.cbt)*cis9n +
                      c(L12.con,L12.cbt)*cis12n +
                      c(L14.con,L14.cbt)*cis14n +
                      c(L16.con,L16.cbt)*cis16n +
                      c(L20.con,L20.cbt)*cis20n

        # common factor (co)variances
        CISpre ~~ 1*CISpre + CISpost
        CISpost ~~ CISpost

        # residual (co)variances
        cis1v ~~ cis1v + cis1n
        cis4v ~~ cis4v + cis4n
        cis6v ~~ cis6v + cis6n
        cis9v ~~ cis9v + cis9n
        cis12v ~~ cis12v + cis12n
        cis14v ~~ cis14v + cis14n
        cis16v ~~ cis16v + cis16n
        cis20v ~~ cis20v + cis20n

        cis1n ~~ cis1n
        cis4n ~~ cis4n
        cis6n ~~ cis6n
        cis9n ~~ cis9n
        cis12n ~~ cis12n
        cis14n ~~ cis14n
        cis16n ~~ cis16n
        cis20n ~~ cis20n

        cis14v ~~ cis20v
        cis14n ~~ cis20n

        # equal intercepts across time (not groups)
        cis1v ~ c(int1.con,int1.cbt)*1
        cis4v ~ c(int4.con,int4.cbt)*1
        cis6v ~ c(int6.con,int6.cbt)*1
        cis9v ~ c(int9.con,int9.cbt)*1
        cis12v ~ c(int12.con,int12.cbt)*1
        cis14v ~ c(int14.con,int14.cbt)*1
        cis16v ~ c(int16.con,int16.cbt)*1
        cis20v ~ c(int20.con,int20.cbt)*1

        cis1n ~ c(int1.con,int1.cbt)*1
        cis4n ~ c(int4.con,int4.cbt)*1
```

```
        cis6n ~ c(int6.con,int6.cbt)*1
        cis9n ~ c(int9.con,int9.cbt)*1
        cis12n ~ c(int12.con,int12.cbt)*1
        cis14n ~ c(int14.con,int14.cbt)*1
        cis16n ~ c(int16.con,int16.cbt)*1
        cis20n ~ c(int20.con,int20.cbt)*1

        # common factor means
        CISpre ~ 0*1
        CISpost ~ 1
'

# run model
CIS.2OUT <- lavaan( CIS.2,
                    data=[DATA],
                    group="[GROUPING VARIABLE]",
                    estimator="MLMV")

# output
summary(  CIS.2OUT,
          fit.measures=TRUE,
          standardized=TRUE)

# test significance with measurement model
anova(CIS.2OUT,CIS.1OUT)



## STEP 3: DETECTION OF SPECIFIC RESPONSE SHIFT

# modification indices
modificationIndices(CIS.2OUT,free.remove=FALSE)

-> include highest MI that indicates response shift
-> test whether response shift is significant in both groups
-> look at parameter estimates to interpret detected response shift

# calculate effect-size of response shift
-> See Verdam, Oort, & Sprangers, 2017 (JCE, 85, 37-44)

## STEP 4: ASSESSMENT OF CHANGE

-> In final model of step 3: look at parameter estimates of means of the underlying latent
factor
-> Compare estimates to the estimates of the no response shift model of step 2)

# calculate effect-size of difference
# Cohen's d: mean_post - mean_pre / sqrt(variance_post + variance_pre -
2correlation_postpre*sd_post*sd_pre)
-> use estimates of the final response shift model to calculate effect-sizes


## STEP 5: INCLUDE EXPLANATORY VARIABLES
```

```
# variables:
# SES (self-efficacy)
# IMQ (focusing on fatigue)
# Fatigue Quality List (Frustrating, Exhausting, Frightening and Pleasant)
# Activity CIS (problems with activity)
# Catastrophising (FCS)

# explanatory variables are included as change-scores

CIS.6 = '

	# equal factorloadings across time (not groups)
	CISpre =~	c(L1.con,L1.cbt)*cis1v +
			c(L4.con,L4.cbt)*cis4v +
			c(L6.con,L6.cbt)*cis6v +
			c(L9.con,L9.cbt)*cis9v +
			c(L12.con,L12.cbt)*cis12v +
			c(L14.con,L14.cbt)*cis14v +
			c(L16.con,L16.cbt)*cis16v +
			c(L20.con,L20.cbt)*cis20v
	CISpost =~	c(L1.con,L1.cbt)*cis1n +
			c(L4.con,L4n.cbt)*cis4n + ## unequal factorloading treatment group
			c(L6.con,L6.cbt)*cis6n +
			c(L9.con,L9.cbt)*cis9n +
			c(L12.con,L12.cbt)*cis12n +
			c(L14.con,L14.cbt)*cis14n +
			c(L16.con,L16.cbt)*cis16n +
			c(L20.con,L20.cbt)*cis20n

	# define all explanatory variables as latent variables
	# include effects on item with detected response shift
	LV.IMQ =~ 1*IMQ + cis4n
	LV.SES =~ 1*SES + cis4n
	LV.FQL_Frus =~ 1*FQL_Frus + cis4n
	LV.FQL_Frig =~ 1*FQL_Frig + cis4n
	LV.FQL_Exha =~ 1*FQL_Exha + cis4n
	LV.FQL_Plea =~ 1*FQL_Plea + cis4n
	LV.CISACT =~ 1*cisact  + cis4n # + c(0,NA)*cis9n
	LV.FCS =~ 1*FCS  + cis4n

	# (co)variances between CISpre and CISpost and all explanatory variables
	CISpre ~~ 1*CISpre + CISpost + LV.IMQ + LV.SES + LV.FQL_Frus + LV.FQL_Frig +
LV.FQL_Exha + LV.FQL_Plea + LV.SES + LV.CISACT + LV.FCS
	CISpost ~~ CISpost + LV.IMQ + LV.SES + LV.FQL_Frus + LV.FQL_Frig +
LV.FQL_Exha + LV.FQL_Plea + LV.SES + LV.CISACT + LV.FCS

	# (co)variances between all explanatory variables
	LV.IMQ ~~ LV.IMQ + LV.IMQ + LV.SES + LV.SES
			+ LV.FQL_Frus + LV.FQL_Frig + LV.FQL_Exha + LV.FQL_Plea
			+ LV.CISACT + LV.FCS
	LV.SES ~~ LV.SES + LV.SES
			+ LV.FQL_Frus + LV.FQL_Frig + LV.FQL_Exha + LV.FQL_Plea
			+ LV.CISACT  + LV.FCS
	LV.FQL_Frus ~~	LV.FQL_Frus
```

```
                 + LV.FQL_Frig + LV.FQL_Exha + LV.FQL_Plea
                 + LV.CISACT + LV.FCS
LV.FQL_Frig ~~ LV.FQL_Frig
                 + LV.FQL_Exha + LV.FQL_Plea
                 + LV.CISACT + LV.FCS
LV.FQL_Exha ~~ LV.FQL_Exha
                 + LV.FQL_Plea
                 + LV.CISACT + LV.FCS
LV.FQL_Plea ~~ LV.FQL_Plea
                 + LV.CISACT  + LV.FCS
LV.CISACT ~~ LV.CISACT + LV.FCS
LV.FCS ~~  LV.FCS

# residual (co)variances
cis1v ~~ cis1v + cis1n
cis4v ~~ cis4v + cis4n
cis6v ~~ cis6v + cis6n
cis9v ~~ cis9v + cis9n
cis12v ~~ cis12v + cis12n
cis14v ~~ cis14v + cis14n
cis16v ~~ cis16v + cis16n
cis20v ~~ cis20v + cis20n

cis1n ~~ cis1n
cis4n ~~ cis4n
cis6n ~~ cis6n
cis9n ~~ cis9n
cis12n ~~ cis12n
cis14n ~~ cis14n
cis16n ~~ cis16n
cis20n ~~ cis20n

cis14v ~~ cis20v
cis14n ~~ cis20n

# equal intercepts across time (not groups)
cis1v ~ c(int1.con,int1.cbt)*1
cis4v ~ c(int4.con,int4.cbt)*1
cis6v ~ c(int6.con,int6.cbt)*1
cis9v ~ c(int9.con,int9.cbt)*1
cis12v ~ c(int12.con,int12.cbt)*1
cis14v ~ c(int14.con,int14.cbt)*1
cis16v ~ c(int16.con,int16.cbt)*1
cis20v ~ c(int20.con,int20.cbt)*1

cis1n ~ c(int1.con,int1.cbt)*1
cis4n ~ c(int4.con,int4.cbt)*1
cis6n ~ c(int6.con,int6.cbt)*1
cis9n ~ c(int9.con,int9.cbt)*1
cis12n ~ c(int12.con,int12.cbt)*1
cis14n ~ c(int14.con,int14.cbt)*1
cis16n ~ c(int16.con,int16.cbt)*1
cis20n ~ c(int20.con,int20.cbt)*1
```

```
        # common factor means
        CISpre ~ 0*1
        CISpost ~ 1
        LV.SES ~ 1
        LV.IMQ ~ 1
        LV.FQL_Frus ~ 1
        LV.FQL_Frig ~ 1
        LV.FQL_Exha ~ 1
        LV.FQL_Plea ~ 1
        LV.CISACT ~ 1
        LV.FCS ~ 1
'

# run model
CIS.6OUT <- lavaan( CIS.6,
                    data=[DATA],
                    group="[GROUPING VARIABLE]",
                    estimator="MLMV")

# output
summary(    CIS.6OUT,
            fit.measures=TRUE,
            standardized=TRUE)
```

-> look at estimates of effects from explanatory variables on item affected by response shift
-> test whether detected response shift is still significant

References

1. Janse A, Worm-Smeitink M, Bussel-Lagarde J, Bleijenberg G, Nikolaus S, Knoop H. Testing the efficacy of web-based cognitive behavioural therapy for adult patients with chronic fatigue syndrome (CBIT): Study protocol for a randomized controlled trial. BMC Neurol. BMC Neurology; 2015;15:1–10.

2. Janse A, Worm-Smeitink M, Bleijenberg G, Donders R, Knoop H. Efficacy of web-based cognitive-behavioural therapy for chronic fatigue syndrome: Randomised controlled trial. Br J Psychiatry. 2018;212:112–8.

3. Abrahams HJG, Gielissen MFM, Goedendorp MM, Berends T, Peters MEWJ, Poort H, et al. A randomized controlled trial of web-based cognitive behavioral therapy for severely fatigued breast cancer survivors (CHANGE-study): study protocol. BMC Cancer. 2015;15:765.

4. Abrahams HJG, Gielissen MFM, Donders RRT, Goedendorp MM, van der Wouw AJ, Verhagen CAHHVM, et al. The efficacy of Internet-based cognitive behavioral therapy for severely fatigued survivors of breast cancer compared with care as usual: A randomized controlled trial. Cancer. 2017;123:3825–34.

5. Menting J, Tack CJ, van Bon AC, Jansen HJ, van den Bergh JP, Mol MJTM, et al. Web-based cognitive behavioural therapy blended with face-to-face sessions for chronic fatigue in type 1 diabetes: a multicentre randomised controlled trial. Lancet Diabetes Endocrinol. 2017;5:448–56.

6. Menting J, Nikolaus S, Wiborg JF, Bazelmans E, Goedendorp MM, van Bon AC, et al. A web-based cognitive behaviour therapy for chronic fatigue in type 1 diabetes (Dia-Fit): Study protocol for a randomised controlled trial. Trials; 2015;16:1–9.

7. Jacobsen PB, Azzarello LM, Hann DM. Relation of Catastrophizing to Fatigue Severity in Women with Breast cancer. Cancer Res Ther Control. 1999;8:155–64.

8. Gielissen MFM, Verhagen CAHHVM, Bleijenberg G. Cognitive behaviour therapy for

fatigued cancer survivors: long-term follow-up. Br J Cancer. 2007;97:612–8.

9. Ray C, Weir W, Stewart D, Miller P, Hyde G. Ways of coping with Chronic Fatigue Syndrome: Development of an illness management questionnaire. Soc Sci Med. 1993;37:385–91.

10. Vercoulen JHMM, Swanink CMA, Fennis JFM, Galama JMD, van der Meer JWM, Bleijenberg G. Dimensional assessment of chronic fatigue syndrome. J Psychosom Res. 1994;38:383–92.

11. Gielissen MF, Knoop H, Servaes P, Kalkman JS, Huibers MJ, Verhagen S, et al. Differences in the experience of fatigue in patients and healthy controls: Patients' descriptions. Health Qual Life Outcomes. 2007;5:1–7.
